# Biotechnological Activities and Applications of Bacterial Pigments Violacein and Prodigiosin

**DOI:** 10.1186/s13036-021-00262-9

**Published:** 2021-03-11

**Authors:** Seong Yeol Choi, Sungbin Lim, Kyoung-hye Yoon, Jin I. Lee, Robert J. Mitchell

**Affiliations:** 1 School of Life Sciences, Ulsan National Institute of Science and Technology (UNIST), Ulsan, 44919 South Korea; 2 Department of Physiology, Mitohormesis Research Center, Yonsei University Wonju College of Medicine, Wonju, Gangwon-do South Korea; 3 Division of Biological Science and Technology, College of Science and Technology, Yonsei University, Mirae Campus, Wonju, Gangwon-do South Korea

**Keywords:** Prodigiosin, Violacein, Antibacterial, Anticancer, Secondary Metabolite, Production, Synthetic Biology

## Abstract

In this review, we discuss violacein and prodigiosin, two chromogenic bacterial secondary metabolites that have diverse biological activities. Although both compounds were “discovered” more than seven decades ago, interest into their biological applications has grown in the last two decades, particularly driven by their antimicrobial and anticancer properties. These topics will be discussed in the first half of this review. The latter half delves into the current efforts of groups to produce these two compounds. This includes in both their native bacterial hosts and heterogeneously in other bacterial hosts, including discussing some of the caveats related to the yields reported in the literature, and some of the synthetic biology techniques employed in this pursuit.

## Introduction

Bacterial strains are capable of producing many different secondary metabolites, including anti-cancer and antibiotic drugs. Here, we discuss two such compounds that are gaining interest due to their diverse biological activities, namely violacein and prodigiosin. Both of these compounds are synthesized by Gram-negative hosts and have been shown in studies from a wide berth of groups to possess important biological activities, including as potent antibiotics against multidrug resistant pathogens. Although both compounds were “discovered” nearly a century ago in the mid-20^th^ century [1–3], their biological activities are still being studied to this day. However, one critical factor limiting research with either compound is their cost, which range from $360 to $760 per milligram [4]. Within this review, therefore, discussion will be given primarily to the biological activities of these compounds, focusing on ecological and medical considerations of both violacein and prodigiosin, as well as current methods to over-produce these remarkable compounds.

## Violacein and Prodigiosin – Hydrophobic Bacterial Chromogenic Pigments

**Fig. 1:**
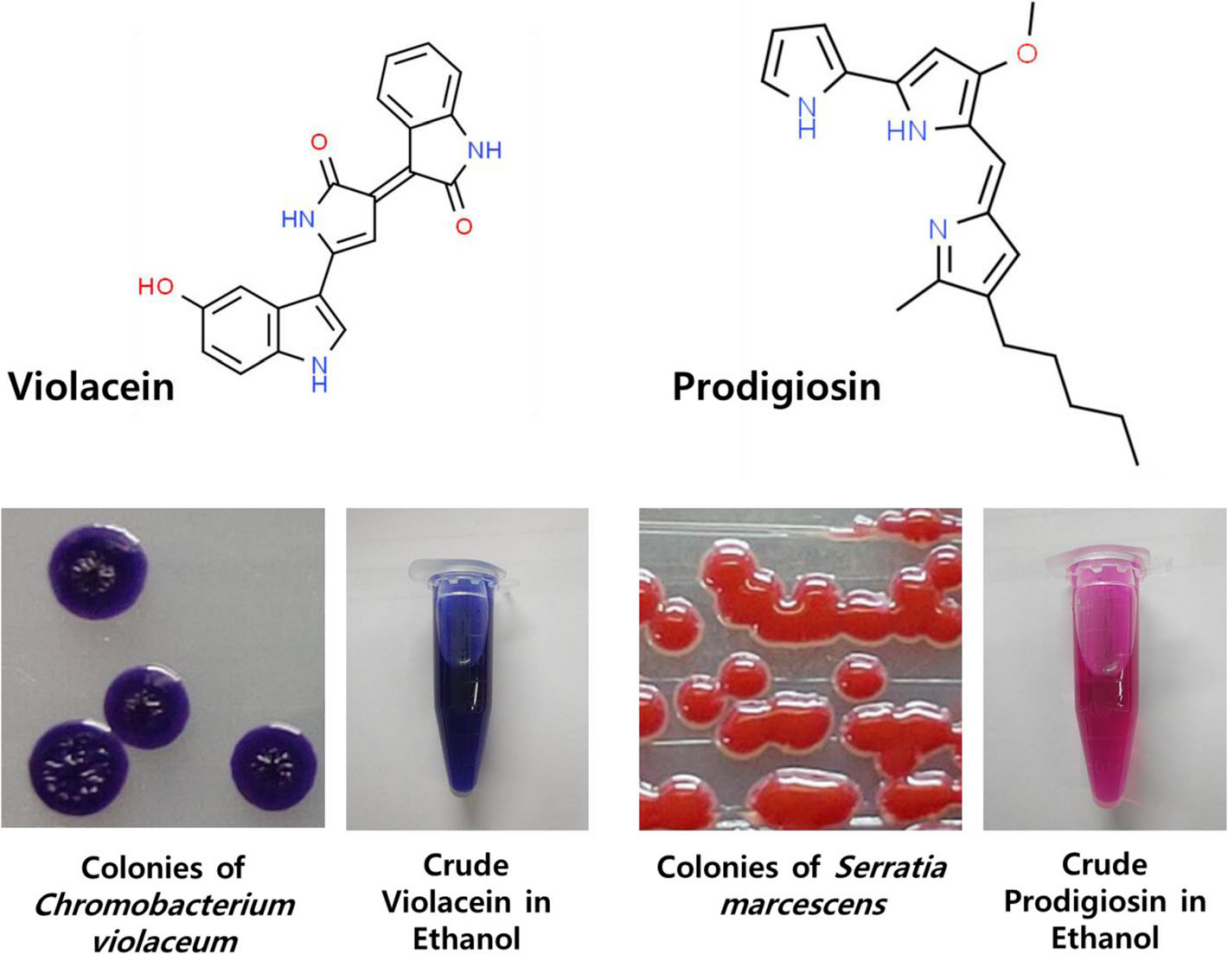
Violacein and prodigiosin, showing the chemical structure and the colored phenotypes of the bacterial strains that produce these compounds

Prodigiosin and violacein are both colorful secondary metabolites, a trait that makes isolating and identifying the bacterial strains that produce these compounds in sufficient quantities easier. As shown in Fig. 1, violacein is a purple-hued bacterial pigment. The fact that this compound is produced by a range of natural bacterial strains [5–8], including *Chromobacterium* [9] and *Janthinobacterium* [10], and in a wide-array of environmental locales, including the deep seas [11], rivers [9, 12], agricultural and forest soils [8, 13, 14], within polar and alpine glacial regions [7, 15, 16], and even on the leaves of white clover [17] and the skin of amphibians [18], all suggest the production of violacein should be relatively advantageous for the host. However, the octanol-water partitioning coefficient (Log P_OW_) for violacein is 3.34 [19, 20], classifying this compound as highly hydrophobic and suggesting it is not readily secreted by the host into the surrounding environment.

Similarly, prodigiosin is vibrant red in color (Fig. 1) and is produced by a number of different Gram-negative and Gram-positive bacterial strains, including *Serratia marcescens* [21] and *Streptomyces*. As a compound, prodigiosin is a member of the prodiginines, a group of chemicals with the same parent nucleus but differing side groups. For this review, emphasis will be given primarily to prodigiosin as this is the most extensively studied compound within this group. When compared with violacein, prodigiosin is even more hydrophobic, with a Log P_OW_ of 5.16 [22].

## Violacein and Prodigiosin as Antimicrobials

The antimicrobial activities of these two compounds have been extensively studied (Tables 1 and 2), particularly for violacein. It is historically recognized that very few Gram-negative bacteria are susceptible to violacein, data that is supported by independent groups in many recent studies [3, 39–41, 58]. The fact that violacein has been produced in recombinant strains of *E. coli*, as well as in *Salmonella typhimurium* VNP20009, *Enterobacter aerogenes* IAM1183 and *Citrobacter freundii* ACCC 05411, with no clear detriment to the growth or viability of these strains [59–62] supports this further. However, individual studies from some groups recently claim violacein exhibits low MIC or growth inhibitory activities with Gram-negative strains [63–65]. Given the historicity and wide range of reports suggesting otherwise, the veracity of these studies needs to be demonstrated independently by other research groups.

**Table 1:** Prodigiosin’s antibiotic activity against microorganisms

Microbe	Description	Reference
**Bacteria**		
Bacillus cereus		[23]
Bacillus subtilis		[24]
Enterobacter cloacae		[25]
Escherichia coli		[23, 25] [26]
Klebsiella aerogenes	Human pathogen	[25]
Pseudomonas aeruginosa	Human pathogen	[25]
Staphylococcus aureus	Human pathogen	[23, 25–27]
Streptococcus pyogenes	Human pathogen	[27]
**Fungi**		
Batrachochytrium dendrobatidis	Amphibian pathogen	[28]
Batrachochytrium salamandrivorans	Amphibian pathogen	[28]
Botrytis cinerea	Plant pathogen	[29]
Fusarium oxysporum	Plant pathogen	[30]
Mucor irregularis	Human pathogen	[31]
Mycosphaerella fijiensis	Plant pathogen	[32]
Phytophthora infestans	Plant pathogen	[30]
Pythium myriotylum	Plant pathogen	[30]
Rhizoctonia solani	Plant pathogen	[30, 33]
Sclerotium rolfsii	Plant pathogen	[30]
**Virus**		
HSV-1	Herpes	[34]
**Protozoa**		
Plasmodium falciparum	Malaria	[35, 36]
Trypanosoma cruzi	Parasitic euglenoids	[37]
**Insect**		
Aedes aegypti	Yellow fever mosquito	[38]
Anopheles stephensi	Malaria vector	[38]

**Table 2:** Violacein’s antibiotic activity against microorganisms

Microbe	Description	Reference
**Bacteria**		
Bacillus anthracis	Anthrax	[3]
Bacillus cereus		[39]
Bacillus licheniformis		[40]
Bacillus megaterium	Plant pathogen	[3, 40]
Bacillus mesentericus	Potential probiotics	[3]
Bacillus subtilis	Common soil bacteria	[3, 40]
Corynebacterium diphtheriae	Diphtheria	[3]
Neisseria meningitidis	Meningococcal disease	[3]
Pseudomonas aeruginosa	Human pathogen	[40, 41]
Staphylococcus aureus	Human pathogen	[3, 39–41]
Staphylococcus epidermidis		[3, 39]
Staphylococcus haemolyticus	Human pathogen	[3]
Streptococcus pneumoniae	Pneumonia	[3]
Viridans streptococci		[3]
**Fungi**		
Aspergillus flavus		[42]
Batrachochytrium dendrobatidis	Amphibian chytrid fungus	[18, 28, 43, 44]
Batrachochytrium salamandrivorans	Amphibian chytrid fungus	[28, 44]
Bipolaris leersia		[45]
Botrytis cinerea	Plant pathogen	[45–47]
Candida albicans	Yeast	[42]
Candida tropicalis	Yeast	[42]
Colletotrichum acutatum	Plant pathogen	[47]
Colletotrichum dematium	Plant pathogen	[45]
Colletotrichum glycines	Plant pathogen	[46]
Colletotrichum orbiculare	Plant pathogen, Affected by deoxyviolacein	[46]
Cryptococcus gastricus		[42]
Diaporthe nomurai		[45]
Fusarium lateritium	Plant pathogen	[45]
Fusarium oxysporum	Plant pathogen	[42, 46]
Fusarium solani	Plant pathogen	[45]
Gibberella zeae	Plant pathogen, Affected by deoxyviolacein	[46]
Magnaporthe grisea	Plant pathogen, Affected by deoxyviolacein	[46]
Penicillium expansum	Plant pathogen	[42]
Phytophthora capsici	Plant pathogen	[46]
Rhizoctonia solani	Plant pathogen, Affected by deoxyviolacein	[42, 46]
Rosellinia necatrix	Plant pathogen	[45]
Saccharomyces cerevisiae	Yeast	[3]
Sclerotinia sclerotiorum	Plant pathogen	[46]
Trichophyton rubrum	Athlete's foot fungus	[42]
Ustilaginoidea oryzae		[46]
Verticillium dahliae	Plant pathogen	[46]
**Virus**		
HSV-1	Herpes	[48]
Poliovirus type 2	Poliomyelitis	[48]
Simian rotavirus SA11	Rotavirus	[48]
**Nematode**		
Bursaphelenchus xylophilus	Pine wilt nematode	[49]
Caenorhabditis elegans		[50, 51]
**Protozoa**		
Acanthamoeba castellanii	Amoeba	[6]
Leishmania amazonensis	Leishmaniasis parasite	[52]
Plasmodium chabaudi	Malaria	[53]
Plasmodium falciparum	Malaria	[53, 54]
Rhynchomonas nasuta		[6]
Tetrahymena sp.		[6]
Trypanosoma brucei gambiense	Human parasite	[55]
Trypanosoma cruzi	Human parasite	[54]
**Insect**		
Drosophila melanogaster	Fruit flies	[56]
Spodoptera litura	Plant pest insects	[57]

In contrast, the activity of violacein against many different Gram-positive bacterial strains (Table 1), including *Staphylococcus*, *Bacillus* and *Streptococcus* [3, 40], is well established. Despite this, its spectrum does not extend to all Gram-positive strains. For instance, *Enterococcus faecalis* ATCC 29212 was not affected by the addition of violacein [66], while *Corynebacterium glutamicum* ATCC 21850 was genetically engineered to produce violacein [67]. It also exhibits antibiotic activities against *Mycobacterium tuberculosis* and *M. smegmatis*, which are acid-fast microbes, and the Gram-variable *Micrococcus luteus* [7, 68].

Stemming from its recognized activities against Gram-positive strains, many recent studies have evaluated the use of violacein against antibiotic-resistant strains of *S. aureus* [8, 41, 58, 66]. For instance, the minimal inhibitory concentrations (MICs) for several *S. aureus* associated with Bovine Mastitis were between 6.25 and 25.00 μM violacein, even though these strains displayed penicillin, ampicillin and/or intermediary erythromycin resistance [58]. Moreover, violacein acted synergistically with penicillin [58], an idea that was expanded on in another study [64]. A separate study using methicillin-resistant *S. aureus* (MRSA) reported MICs in basically the same range, *i.e.*, 7.5 to 30 μM [66], while research from our group found a multidrug-resistant *S. aureus* clinical isolate with resistance to seven different antibiotics was also susceptible to violacein [8]. In that study, the MICs for both the clinical isolate and the non-resistant type strain (*S. aureus* ATCC 25923) were identical (15 μM) while bactericidal effects against both were seen when 30 μM or more violacein was employed [8]. This proved the antibacterial mechanism used by violacein differs from that of the other antibiotics and also that cross-resistance was not present.

For both compounds, their antimicrobial activities stem in part due to their lipophilic natures. When introduced into a bacterial culture, prodigiosin and violacein rapidly insert into the membranes of the microbe and disrupt their integrity, leading to ATP and protein leakage [22, 69, 70]. Interactions between violacein and bacterial membranes were recently modeled [70], and suggested that this compound does not embed very deeply within the lipid bilayer. The same study looked at the release of carboxyfluorescein from large unilamellar vesicles (LUVs) prepared using the lipids from three different bacteria, *i.e.*, *E. coli* ATCC 25922, *B. subtilis* PY79 and *S. aureus* ATCC 25923. They found, regardless of the strain, the LUVs were equally susceptible [70], implying *E. coli* cellular membranes are just as likely to be attacked by violacein and that its inherent resistance to violacein stems from the protective nature of the outer membrane, which absorbs this antibiotic and prevents its access to the cytoplasmic membrane. Recent work from our group studied this further, but from a different perspective, by asking how violacein acts as an antibiotic in nature if it is hydrophobic and remains embedded primarily within the membrane of the strain that produced it. It was found *C. violaceum* secretes violacein within membrane vesicles (MVs) [20]. These vesicles bud off of the bacterium as it grows and contained more violacein than proteins (mg/mg), increasing the apparent water solubility of violacein. Using *S. aureus* and a violacein-deficient *vioA* mutant, the violacein-carrying MVs were proven to be bactericidal, although a greater overall amount of violacein was required to achieve the same killing efficiencies as crude purified violacein. In contrast, MVs from the *vioA* mutant had no impact on *S. aureus* viabilities, proving violacein was the bactericidal factor responsible.

A recent study also performed molecular dynamic simulations with prodigiosin [71]. The authors found, in contrast to violacein, prodigiosin embedded itself much deeper within the membrane lipid bilayer, a finding that helps explain why this compound is effective against some Gram-negative strains as this would increase the chances for prodigiosin to penetrate the outer membrane and enter the cytoplasmic membrane. However, it still remains to be seen if MVs are also used by prodigiosin-producing strains to transport this antibiotic to susceptible microbes.

In addition to membrane disruption, prodigiosin apparently causes additional damage within the bacterium, including the generation of reactive oxygen species (ROS) [23, 72] and, based on the study by Darshan and Manonmani (2016) [23], interacting with the bacterial genomic DNA. This latter facet of its activities corroborates an earlier study where prodigiosin was shown to cleave double-stranded DNA *in vitro* [73], an activity that is mediated by oxidative radicals (*i.e.*, ROS) and requires the presence of a redox-active transition metal since the addition of either catalase or EDTA inhibited cleavage. Taken together, both studies suggest the ROS production by prodigiosin and its interactions with redox-active transition metals may act in concert *in vivo* to cause DNA damage within the bacterial cell, although this would benefit from further verification.

## Prodigiosin and Violacein as Antifungals

In addition to their application towards bacterial pathogens, violacein (and its deoxyviolacein derivative) and prodigiosin also work widely and effectively against many pathogenic fungi (Tables 1 and 2). For violacein, representative examples of fungi that are susceptible include the plant pathogen *Rhizoctonia solani* [42, 46] and *Batrachochytrium dendrobatidis* [43, 44], a fungus that is lethal to amphibians. In the latter case, the presence of a violacein-producing bacterium, *J. lividum*, on the skin of the black-backed salamander (*Plethodon cinereus*) [44] or frog (*Rana muscosa*) [43] provided protection against *B. dendrobatidis*. Under these conditions, this bacterium was clearly able to produce a significant amount of violacein as the skin-associated concentrations with the frogs averaged around 100 μM, which was much higher than the 18 μM MIC needed to prevent mortality and morbidity caused by *B. dendrobatidis* based on the salamander study.

Although not studied as extensively, several reports have also discussed prodigiosin and its activities against different fungal species [30, 74–77]. Much like the two studies mentioned above, one group even looked at the ability of *S. marcescens* to protect *Acris blanchardi* (Blanchard’s Cricket frog) from *B. dendrobatidis* infections, reporting a slight, yet significant, increase in survival rates when compared against a *pig* mutant that is unable to synthesize this compound [77]. Moreover, although the mechanism of action is not fully understood, detailed observation of *S. marcescens* invading into fungus was reported recently [31]. In that study, prodigiosin increased the membrane permeability of target cell, enabling *S. marcescens* to invade into *F. oxysporum*. Given prodigiosin’s ability to damage the target cell’s membrane was also suggested as a mechanism of action against other bacterial cells [22], it would appear this compound has similar properties against organisms spanning different kingdoms.

## Violacein and Prodigiosin as Nematicidal and Anti-Protozoan Agents

A benefit of violacein and prodigiosin for the producing bacteria is that it confers a survival advantage against competitors and predators, providing selective advantages against neighboring bacteria and an effective defense and deterrent against bacterivores, such as protozoa and nematodes (Tables 1 and 2).

Nematodes have caused detrimental disease to both humans and agriculture worldwide. Pine wilt disease, a serious epidemic that has devastated pine forests globally, especially in East Asia, is caused by the nematode *Bursaphelenchus xylophilus*. This nematode, also called pine wilt nematode, attacks the water transport system of pine trees, causing them to wilt and die [78]. Expensive nematicides have commonly been used to combat pine wilt nematode with little success. Recently, a violacein5'-O-glucoside derivative was constructed by expressing the glycosyltransferase (*YjiC*) from a *Bacillus* sp. in *E. coli* along with the *vioABCDE* [49]. This novel violacein derivative had increased water solubility and was an effective treatment against the pine wood nematode [49], suggesting its potential use in the future as an anti-nematodal agent against pine wilt disease.

**Fig. 2:**
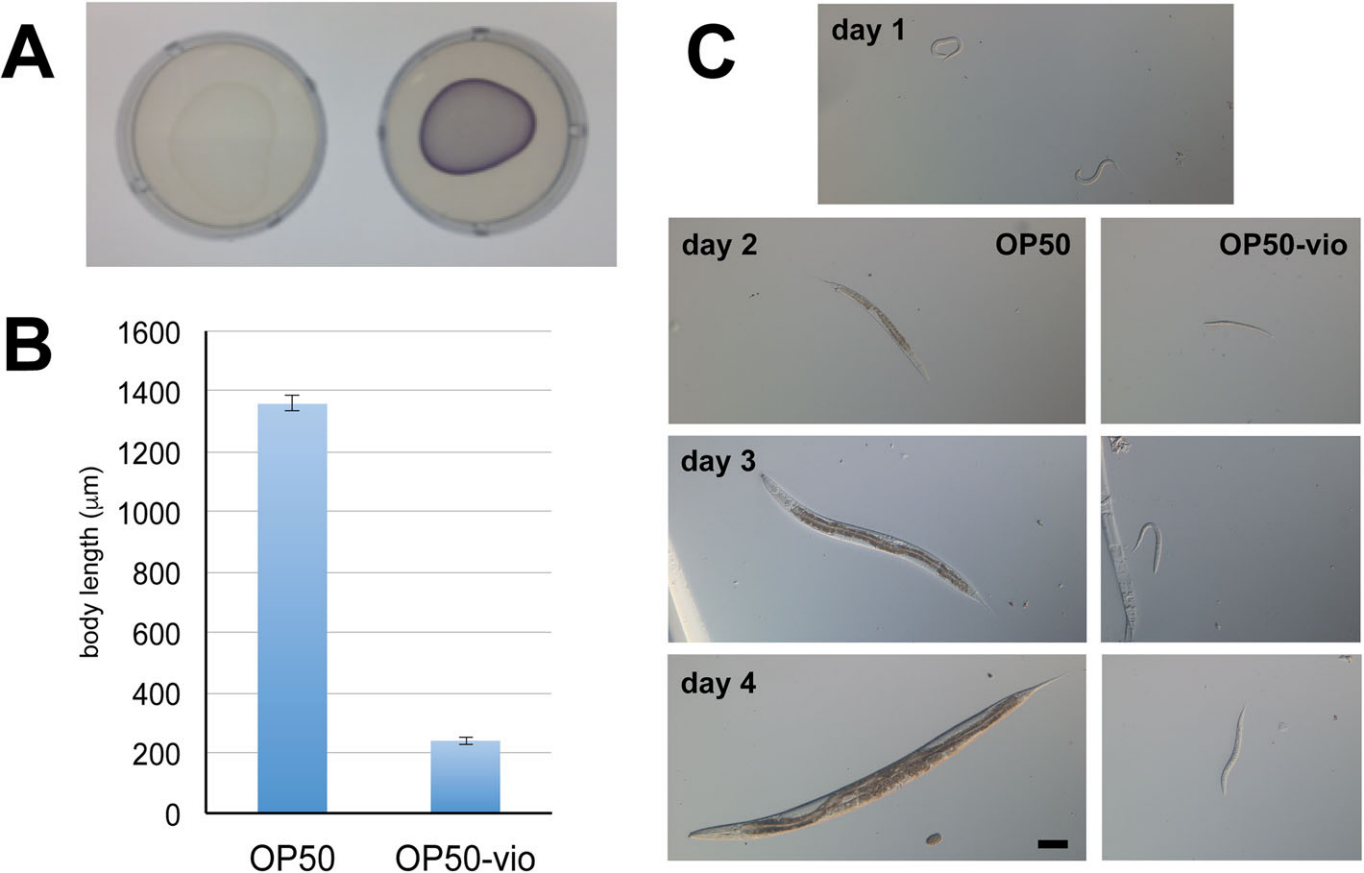
Violacein stunts the growth and development of *C. elegans*. (A) Lawn of *E. coli* strain OP50 (left) and violacein-expressing OP50 (OP50-vio, right). (B) Body length of worms grown on OP50 and OP50-vio from L1 larvae stage for 4 days. (C) Development of worms grown on OP50 and OP50-vio. Day 1 image show L1 synchronized worms that has never been fed. Scale bar = 100 μm. Figure originally published in [79]

Violacein also negatively impacted the nematode genetic model organism *C. elegans*. When fed on violacein-producing *Janthinobacterium*, *C. elegans* displayed developmental arrest in early larval stages [50]. Similar developmental arrest and delay was seen when violacein was expressed in *E. coli* OP50 [79] (Fig. 2)*,* the normal laboratory diet of *C. elegans* [50]. Consumption of this compound induced the expression of several detoxification genes regulated by the insulin-like signaling pathway [80]. Interestingly, supplementation of unsaturated fatty acids, especially oleate, alleviated the worm growth and survival in violacein, whereas saturated fatty acids had no effect [79]. In addition to highlighting the anti-nematodal potential of violacein, studies in *C. elegans* may help also elucidate if a conserved mechanism of violacein-induced toxicity in metazoans exists. With the extensive genetic and molecular tools available for *C. elegans*, exploring how unsaturated fatty acids are able to mitigate violacein’s toxicity may provide a window into this mechanism, and may also shed light on its activities within cancer cells.

## Anticancer Activities of Prodigiosin and Violacein

Another well-known characteristic of these two compounds is their anti-tumor activities. Cancer is the second leading cause of death globally [81], and although recent therapeutics have been developed for some cancers, still it remains as devastating as ever. In the laboratory, prodigiosin has been reported to kill human cancer cell lines by a process called programmed cell death or apoptosis. Prodigiosin can induce apoptosis in haematopoietic cancer cells [82], human lung cancer cells [83], B cells and T cells in chronic lymphocytic leukemia [84], gastric cancer cells [85], multidrug resistant breast cancer cells [86], colorectal cancer cells [87] and glioblastoma multiforme cancer cells [88] (Table 3).

**Table 3:** List of cell lines evaluated with prodigiosin

Cell Line	Description	References
95-D	Human highly metastatic lung cancer	[89]
B-CLL	Chronic lymphocytic leukemia	[84]
DLD1	Colorectal cancer	[87]
GLC4	Small cell lung cancer	[83]
A549	Lung cancer	[90]
HCT116	Colorectal cancer	[87, 91]
SW480	Colorectal cancer	[87, 91]
SW620	Colorectal cancer	[87]
HGT-1	Gastric cancer	[85]
HL-60	Haematopoietic cancer	[73, 82]
Jurkat	Haematopoietic cancer	[82, 92]
U87MG	Glioblastoma cancer	[88]
GBM8401	Glioblastoma cancer	[88]
MCF-7	Breast cancer	[86, 92]
MDA-MB-231	Breast cancer	[86, 93]
NSO	Haematopoietic cancer	[82]
Ramos	Haematopoietic cancer	[82]

Despite the strong evidence that prodigiosin can work against multiple types of cancer cells, how this compound targets cancer cell death by apoptosis is not yet clear. Prodigiosin can interact with and cleave DNA [73, 92], supporting one possible mechanism of cell death. Prodigiosin also facilitates proton and chloride ion symport and can affect the acidification of cellular compartments [94, 95], providing support for an alternative mechanism of cancer cell apoptosis [90]. Finally, prodigiosins also inhibit protein phosphatase activity *in vitro* [96, 97], suggesting another possible mechanism of how this compound may inhibit cancer cell growth.

More recent studies have suggested that prodigiosin causes cell death by affecting a cellular process called autophagy. The process of autophagy causes an accumulation of specific vesicles in the cell called autophagosomes that can break down damaged organelles or proteins [98]. Autophagy has also been a target for cancer therapy [99], especially due to the fact that this cellular process also regulates apoptosis in cancers [100]. In a recent laboratory study, prodigiosin treatment induced the death of glioblastoma cancer cells and reduced neurosphere growth, a marker associated with increased death in glioblastoma patients [88]. The authors further showed that apoptotic death of the glioblastoma cells by prodigiosin treatment was due to increased autophagy in the cancer cells. In another recent study, colorectal cancer cells that were treated with the chemical 5-fluorouracil, a common chemotherapy treatment for colorectal cancer, showed increased apoptosis in the presence of prodigiosin [91]. Interestingly, prodigiosin impaired autophagic flux which actually promoted cell death in the cancer cells in response to 5-fluorouracil.

Combination therapy, which uses two or more therapeutic agents as a cancer treatment, has become a main strategy in cancer therapy in recent years [101]. The use of prodigiosin in combination with other cancer therapies is a promising strategy that is currently being explored. As mentioned previously, 5-fluorouracil in combination with prodigiosin effectively killed colorectal cancer cells by increasing apoptosis [91]. In addition, a recent study showed that the combination of prodigiosin and PU-H71, a candidate therapy for triple negative breast cancer, induced apoptosis in a metastatic breast cancer cell line killing many of the cancer cells [93]. These studies, as well as others, confirm that prodigiosin promotes the killing of cancer cells in the laboratory and demonstrate that it is an excellent candidate for cancer therapy either as a combination therapy or singular treatment. However, whether this activity can actually translate to a treatment for cancer patients remains unknown. Several phase I and phase II clinical studies with various cancer patients have occurred with a prodigiosin derivative called obatoclax [102–105], and the jury is still out on whether prodigiosin is an effective therapy for human cancer patients.

Similar with prodigiosin, violacein is also a promising anti-tumor bacterial metabolite (Table 4). As with prodigiosin, violacein leads to mitochondrial dysfunction, brought on by mitochondrial membrane hyperpolarization, in MRC-5 and HeLa cells [111]. It was also confirmed in RAS-mutated metastatic melanoma cell lines that the autophagy process employed to resolve mitochondrial damage is impaired due to inhibition of AKT and AXL [115]. Subsequent processes followed a general apoptotic pathway leading to p38 MAP kinase phosphorylation, NFκB pathway activation, and activation of caspases when treated with 1 μM of violacein in HL60 [113]. However, in TF1, which is known to have apoptosis resistance, the IC50 was still only 2 μM despite co-treatment with inhibitors of pro-apoptotic caspases, leading the authors to conclude that violacein induces cell death via the activation of a non-canonical mechanism of cell death [116]. Interestingly, an *in vitro* study showed that violacein inhibits PKA and PKC activity [117]. While the results do not exclude other possible targets, and whether this leads to cancer cell death in vivo awaits to be examined, it suggests PKA and PKC could be a direct target of violacein.

**Table 4:** List of cell lines evaluated with violacein

Cell Line	Description	Ref
92.1	Uveal melanoma	[106]
A549	Lung cancer	[60, 107]
A431	Skin cancer	[60]
Caco-2	Heterogeneous epithelial colorectal adenocarcinoma	[108, 109]
CAL-27	Head and neck carcinoma cells	[110]
CHO-K1	Chinese Hamster Ovary cells	[111]
DLD1	Colorectal adenocarcinoma	[109]
EAT	Mouse Ehrlich ascites tumor	[112]
FaDu	Head and neck carcinoma cells	[110]
FRhK-4	Fetal kidney	[48]
HCT116	Colorectal adenocarcinoma	[60, 109]
HeLa	Hela cell, Cervix cancer	[60, 111]
Hep2	Hela-derived	[48]
HL60	Promyelocytic leukemia	[113]
HN5	Head and neck squamous cell carcinoma cells	[60]
HT29	Colorectal adenocarcinoma	[60, 108]
K562	Lymphoma	[113]
KM12	Colon cancer	[114]
MA104	Monkey Kidney epithelial cells	[48]
MCF7	Breast cancer	[60, 107]
MOLT-4	Acute lymphoblastic leukemia	[114]
MRC-5	Fetal lung fibroblast	[111]
NCI-H460	Non-small-cell lung cancer	[114]
OCM-1	Choroidal melanoma	[106]
PC3	Prostate cnacer	[60]
SALTO	Head and neck carcinoma cells	[110]
SCC-15	Head and neck carcinoma cells	[110]
SKMEL-103	RAS-mutated metastatic melanoma	[115]
SKMEL-28	RAS-mutated metastatic melanoma	[115]
SW480	Colorectal adenocarcinoma	[109]
TF1	Erythroleukemia	[116]
U87	Glioblastoma	[107]
U937	Chronic myelogenic leukemia	[113]
V79	Chinese Hamster Fibroblast-like cell line from lung tissue	[114]
Vero	Monkey Kidney	[48]

This sequence of cell death mechanisms resulting from mitochondrial damage brought on by violacein is due to the profound threat to the energy metabolism of cells. As a good indication of this, violacein has enhanced anti-cancer activities against some cell lines in hypoxia, such as HCT 116 (4.8-fold), HN5 (6.5-fold), HT29 (12.6-fold), and MCF7 (4-fold) [60]. Moreover, violacein treatment (1μM) led to the downregulated expression of chemokine/receptor CXCL12/CXCR4, which is important for angiogenesis [118]. Since carcinoma development without angiogenesis leads to hypoxic conditions, these results suggest violacein may actually induce the conditions within the tumor that increase its effectiveness as an anticancer agent, as was reported in one study [119].

Other studies have confirmed that oral administration of violacein contributes to NSAID-induced gastric damage healing. This led to a decrease in inflammatory cytokines, particularly TNF-α, and an increase in epidermal growth factor (EGF), vascular endothelial growth factor (VEGF) and hepatocyte growth factor (HGF) [120]. These appear to play an essential role in healing angiogenesis and mucin secretion. In other words, violacein administered orally plays a role in inhibiting inflammation, maintaining the balance of cytokines, while also inhibiting apoptosis, angiogenesis, and promoting healing.

## Immunomodulatory Activities of Prodigiosin and Violacein

Prodigiosin is also known to have immunosuppressive effects. Specifically, this compound shows suppressive effects on T-cell proliferation, while having no effect in B-cells [121]. Its mechanism of action is to inhibit expression of the interleukin-2 receptor α(IL-2Rα) chain, an important contributor of T-cell activation [122]. In another study, the authors developed a prodigiosin-analogue molecule, PNU156804, which suppressed both T-cell and B-cell activation [123]. This compound also worked through inhibiting IL-2 dependent signaling, *i.e.*, not by preventing IL-2Rα induction but rather by preventing activation of AP-1 and NF-κB. Prodigiosin was also synergistically active when administered with cyclosporine A, each working through different pathways to suppress T-cell activation [124], while another study found it inhibited macrophage and NK killer cell activities and splenocyte proliferation [125]..

Violacein was also shown to have immunomodulatory functions and inhibit inflammation. For instance, this compound had antipyretic, analgesic, and immunomodulatory reactions when orally administered to rats [126]. In ulcer rat models, violacein relieved inflammation of the gastrointestinal tract, possibly working through the COX-1 mediated pathways [120], while another study reported that, when injected directly into the intraperitoneal cavity, violacein can have immunomodulatory effects by regulating cytokine production: it down-regulated the expression of IL-6 and TNF-α but induced expression of IL-10 [127].

Some of the immunomodulatory mechanisms and findings associated with violacein seem contradictory with the cancer studies, however. Unlike the above study that reported violacein inhibits TNF-α expression [127], TNF-α expression was elevated in HL60, and TNF receptor 1 signaling was also activated when this cell line was exposed to violacein [113]. It is also known to increase the expression of TNF-α and upregulate the p53-dependent mitochondrial pathway in MCF-7 [128], while treatment with violacein also induced TNF-α expression in Raw 264.7 and ANA-1 cells [129]. These differences may be due to the experimental protocols, though, as the above studies were performed *in vitro*, *i.e.*, violacein treatment directly into human or murine cell cultures [128, 129], rather than *in vivo*, *i.e.*, the oral administration or injection of violacein into the digestive tract or intraperitoneal cavity [120, 126, 127]. In other words, vastly different results may result depending on the method of administration and the type of cells, but all of the above studies confirmed that violacein has immunomodulatory aspects.

## Bioproduction - Measurement of Prodigiosin and Violacein – Spectrophotometry vs. HPLC

The classical method for prodigiosin extraction from the bacterial host and culture is to use acidified ethanol (4% 1M HCl v/v) to prevent the rapid decomposition of this molecule when above pH 5. The impurities present in the extracted prodigiosin are then removed using a solvent such as dichloromethane or n-hexane:chloroform and the final product purified through chromatography [130–132].

The simplest way to measure the extracted prodigiosin is to use a spectrophotometer using an absorption wavelength of 530-540 nm and convert this to the concentration using an extinction coefficient (ε) and the Beer–Lambert law. However, this is not without issue as the value of ε varies from study to study. Traditionally, the value of ε535 is 0.159 L/mg-cm [133]. The most detailed study on the extinction coefficient of prodigiosin is Domröse et al. (2015) [130], where ε535 was calculated to be 0.4322 L/mg-cm in acidified ethanol, a value that was confirmed through quantitative 1H-NMR. This value is near identical with that reported by another group, *i.e.*, ε535 = 0.4311 L/mg-cm [134]. Consequently, due to the difference in the extinction coefficients, the prodigiosin concentration using the classical ε value will be over-estimated by 270%.

Similarly, violacein has often been quantified using a spectrophotometer and its absorbance peaks at 575-590nm [8, 135–137]. However, because of differences in reported ε between research groups, the yields claimed in the literature are inconsistent. For example, the ε values for violacein include, from lowest to highest, ε570 = 10.955 L/g-cm in ethanol [138], ε = 29.700 L/g-cm [137], ε565 = 31.3 L/g-cm in acetone-water [139], ε570 = 46 L/g-cm in ethanol [67], ε575 = 56.010 L/g-cm in ethanol [135] and ε575 = 74.3 L/g-cm in ethanol [140]. This disparity was raised in the study by Rodrigues et al. (2013) [140] and in previous reviews [141, 142], potentially inflating the violacein yields by as much as 670%.

To address this issue, Rodrigues et al. (2013) [140] elected to quantify violacein through HPLC [140], a protocol that has been successfully applied within several of our own studies [20, 143, 144]. At present, similar protocols have not been applied to quantify prodigiosin and HPLC may consolidate the yields in the literature, an idea that should be evaluated further. However, given the wide-spread problems raised by this issue, the concentrations of these two compounds reportedly produced in the literature will not be discussed, but rather the qualitative results of the studies.

## Production by Natural Isolates

As discussed above, a wide-range of natural bacterial strains are capable of synthesizing violacein and prodigiosin. It should come as no surprise, therefore, that researchers have sought out a variety of strains for the lab scale production and application of these two compounds. For instance, *S. marcescens* FZSF02 was isolated from the soil in the region of Fuzhou, China, and is capable of producing prodigiosin in sufficient quantities that it reportedly pellets out of solution [145]. Another natural strain, *S. marcescens* MO-1 was isolated from a grasshopper [146] while *S. marcescens* UCP1459 and *S. marcescens* UTM1 were isolated from semi-arid soil in Brazil and an oxidation pond in Malaysia, respectively [147, 148]. A related bacterium, *S. rubidaea*, also produced prodigiosin and was initially isolated from a spoiled coconut, where it was discovered since it changed the color of the inside of the coconut, making it pink [149].

Similarly, violacein production has been studied in different natural strains. For instance, production of this compound in *C. violaceum* CCT 3496 was increased around 2.5-fold when tryptone and yeast extract were added, but the yields dropped with glucose [135]. In a separate study, optimization in *Duganella* sp. B2 found tryptophan, beef extract, and potassium nitrate were all major factors impacting violacein yields [138] while in *Massilia* sp. EP15224, an isolate known to be closely related to *Duganella sp.*, the MM2 broth used to cultivate this strain was improved by adjusting the amount of phosphate, leading to faster production rates and slightly better final violacein yields [150].

Some violacein-producing bacteria are also psychrotrophic, such as strain RT102, which is related with *J. lividum*, reported by Nakmura *et al* (2003) [40]. The authors found that the conditions leading to optimum production levels were a slightly acid pH of 6, the growth temperature set to 20°C and with 1 mg/L of dissolved oxygen. Although not as psychrophilic as RT102, *J. lividum* was also successfully used to produce violacein, albeit at 25 °C and a pH of 7.0 [65]. Notably, in this study, the addition of 0.2 mg/mL of the antibiotic of silver ampicillin improved the yields by a factor of 1.3 while glycerol was used as a carbon source, a choice the authors claimed improves the violacein production relative to the cell mass.

The idea of using ampicillin and glycerol to increase violacein yields was actually reported more than a decade earlier in the study by Pantanella *et al* (2007), where glycerol enhanced violacein production levels by approximately 12-fold, while ampicillin led to an estimated 3-fold increase [136]. These factors, unfortunately, were not additive when used together – the maximum level with glycerol with or without ampicillin were basically identical.

The use of more natural feed stocks was also considered, as in the case with *C. violaceum* UTM5 where agricultural wastes were used [151], or in a separate study where liquid pineapple waste was used as the carbon source along with addition of L-tryptophan [41]. However, as noted above, since these papers do not provide the extinction coefficient and did not use HPLC techniques when quantifying their yields, it is difficult to directly compare their results with other studies.

## Random Mutations to Enhance Prodigiosin Production

One strategy used by researchers to enhance production of prodigiosin is to generate random mutations within the genome of the natural host, typically with radiation. Since prodigiosin is a red pigment, screening is a simple and quick method for researchers to identify those colonies that overproduce this compound based on their color intensity. This was successfully used by one group with microwave irradiation to increase the prodigiosin yields from *S. marcescens* jx1 by just over two-fold [26], while a separate group used gamma irradiation [152]. In the latter study, the authors varied more than just the radiation dose and rate, including the pH and inoculum size, to identify conditions that optimize for prodigiosin production. However, as in the microwave radiation study, the yields were only improved by about 2-fold.

## Heterogeneous Expression and Metabolic Pathway Engineering to Increase Prodigiosin and Violacein Yields

The above yields, although definitely improved, are not very significant and highlight potential limitations linked with random mutation studies, namely that improvements may not be very substantial, particularly when they involve complex metabolic pathways encoded in multiple genes such as those involved in prodigiosin biosynthesis. As such, researchers have often sought to clone and express the genes in other hosts where the metabolic and biosynthetic pathways can be engineered.

The prodigiosin gene cluster (*pig*) includes many genes, *pigA* to *pigN*, but may vary in gene order as well as include some auxiliary genes depending on the bacterial host [153, 154]. During the mid-20th century, studies in prodigiosin biosynthesis focused on related molecular components and constructing the pathway [21, 155–157], including the role of quorum sensing mechanisms [158–161], while recent studies have provided a more detailed understanding of the biosynthetic pathways involved [153, 154]. Violacein research has followed a similar path, with the biosynthetic pathway first mapped in 1991 [162] and the roles of the individual genes and enzymes characterized further in the early 2000’s [163, 164]. In addition, during the same period many articles, were published discussing the roles of quorum sensing in the production of this metabolite [165–167]. This led to the eventual development and application of *C. violaceum* CV026 as a quorum-sensing reporter strain, as it visually responded to the presence of acyl homoserine lactones (AHLs) with the production or inhibition of violacein synthesis [166, 168, 169]. Recently, this strain was reclassified as *C. subtsugae* [170].

As the last two decades have seen sequencing techniques and comparative genome analyses dramatically improve, a new era of prodigiosin and violacein production has opened. Gene clusters related with prodigiosin production were sequenced, analyzed and compared among different species and subspecies [171, 172], as have the genomes of numerous violacein-producing bacteria [173–175], particularly by Dr. Brooke Jude at Bard University who, in the last couple of years, has published several genomes [176–179]. Of particular note, one of the *Janthinobacter* sp. sequenced by her group actually lacked the genes for violacein but carried the *pig* gene cluster, allowing it to produce prodigiosin [180]. They concluded that, since this strain was isolated from the region where other violacein-producing strains were also located, including other *Janthinobacter* sp., the production of prodigiosin by this strain may represent a combined effort by the two groups to combat other bacterial species.

All of this information will aid researchers in further efforts to clone and express the genes required in other bacterial strains. This is not to say that this has not been done already, as a few groups reported the heterogeneous production of prodigiosin [130, 181], one as far back as 1984 [182]. However, only one study truly sought to use the new host, in this case *Pseudomonas putida* KT2440, as a platform for the production of this compound [130]. In their study, the authors introduced the *pig* cluster randomly into the genome of *P. putida* using a plasmid bearing a transposon and screened the resulting clones for prodigiosin production, looking for insertions where the cluster was expressed by a strong promoter. Using this method, they were able to increase prodigiosin production on agar plates by approximately five-fold over the original *S. marcescens* and as much as 94 mg/L, based on their quantification methods, in liquid cultures.

In contrast, the expression of violacein in other bacterial hosts is widespread, with the *vioABCDE* genes cloned and expressed within many plasmids and bacterial hosts. Some examples of this include *Citrobacter freundii* [61, 62], *Klebsiella aerogenes* (formerly *Enterobacter aerogenes*) [62] and *E. coli* [62, 140, 162, 183–185]. Other studies have sought to improve on the violacein yields through synthetic biology, often with *E. coli* as the host [54, 186], albeit not always for purification, as illustrated in two recent studies where its expression was used as a bioreporter [187, 188]. One prime example where synthetic biology was employed to improve violacein production is the study by Jeshek *et al.* (2016) where they introduced the Reduced Libraries algorithm [189]. These used this system to design smart combinatorial libraries for pathway optimization based on the ribosomal binding sites and, in this case, focused on increasing violacein production while minimizing that of deoxyviolacein. A second group used a different approach and elected to express each gene independently by their own promoter [59]. By controlling the strengths of each individual promoter, and using a combinatorial assembly of the genes, they were able to increase the violacein titers by more than 60-fold over the control, where each gene was expressed under the T_7_ promoter. In addition to *E. coli*, other hosts have been used for the heterogeneous production of violacein, including yeasts [190, 191]. One such study used *Yarrowia lipolytica*, an oleaginous yeast, as the host, where the *vio* genes were expressed using three different promoters and assembled using the Golden Gate assembly method to build combinatorial pathway libraries [191]. From this, three yeast strains, each producing a different chromogenic compound, *i.e.*, violacein, deoxyviolacein and proviolacein, were constructed.

## Conclusions

**Fig. 3:**
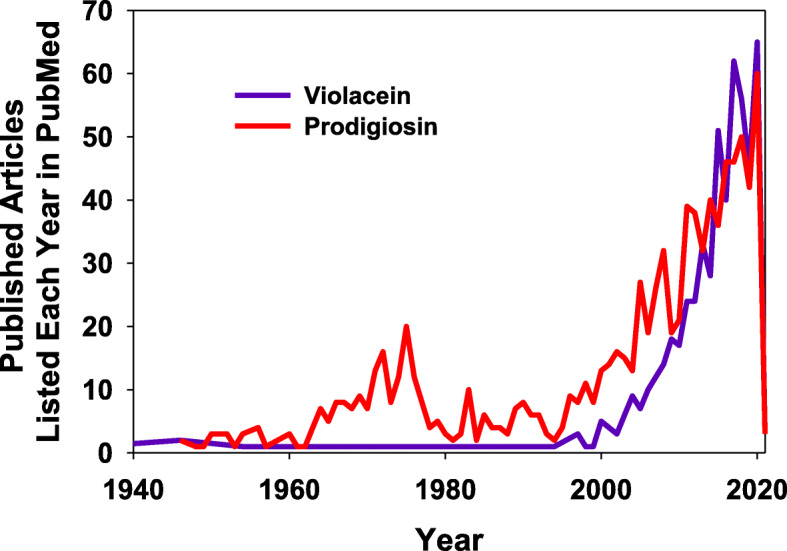
Number of research articles related with prodigiosin and violacein published each year according to the data available at the NCBI PubMed website [192]. (Accessed Jan 20^th^, 2021)

This review presented many biological traits of both prodigiosin and violacein reported in the recent and current literature. Fig. 3 is a plot showing the number of peer-reviewed articles listed in the National Center for Biotechnology Information’s PubMed website [192] for each year, providing visual evidence of the growing interest into these two compounds and their activities. Although the numbers may not be as great as some other hot-topics, the data makes it clear that many research groups continue to study and explore the biological activities of these two compounds and different methods for producing them in greater quantities. As this field continues to expand and mature, other derivatives of violacein and prodigiosin are expected to move towards clinical trials as antimicrobials and for the treatment of human diseases, including cancer, as was noted above for obatoclax. This will be supported in no small part by synthetic biologists and chemical engineers who are currently developing novel and more efficient protocols and strains to increase the productivity and yields of these two secondary metabolites, a trend that is also expected to reduce the costs of these compounds, which at present are too high for conventional medical research.

## Data Availability

None.
